# Comparative Molecular Characterization of Novel and Known Piscine Toti-Like Viruses

**DOI:** 10.3390/v13061063

**Published:** 2021-06-03

**Authors:** Liv Sandlund, Sunil K. Mor, Vikash K. Singh, Soumesh K. Padhi, Nicholas B. D. Phelps, Stian Nylund, Aase B. Mikalsen

**Affiliations:** 1 Pharmaq Analytiq, 5008 Bergen, Norway; liv.sandlund@zoetis.com (L.S.); stian.nylund@zoetis.com (S.N.); 2 Veterinary Diagnostic Laboratory, Department of Veterinary Population Medicine, College of Veterinary Medicine, University of Minnesota, St. Paul, MN 55108-6074, USA; kumars@umn.edu (S.K.M.); vsingh@umn.edu (V.K.S.); 3 Department of Fisheries, Wildlife and Conservation Biology, College of Food, Agriculture and Natural Resource Sciences, University of Minnesota, St. Paul, MN 55108-6074, USA; spadhi@umn.edu (S.K.P.); phelp083@umn.edu (N.B.D.P.); 4 Department of Paraclinical Sciences, Faculty of Veterinary Medicine, Norwegian University of Life Sciences, 1432 Ås, Norway

**Keywords:** piscine, toti-like viruses, *Totiviridae*, phylogeny, genomic characterization

## Abstract

Totiviridae is a virus family well known to infect uni-cellular organisms like fungi and protozoa. In more recent years, viruses characterized as toti-like viruses, have been found in primarily arthropods, but also a couple in planarians and piscine species. These toti-like viruses share phylogenetic similarities to totiviruses; however, their genomes also includes additional coding sequences in either 5′ or 3′ ends expected to relate to more advanced infection mechanisms in more advanced hosts. Here, we applied next generation sequencing (NGS) technologies and discovered three new toti-like viruses, one in wild common carp and one in bluegill from the USA and one in farmed lumpsucker from Norway. These are named common carp toti-like virus 1 (CCTLV-1), bluegill toti-like virus 1 (BGTLV-1), and *Cyclopterus lumpus* toti-like virus (CLuTLV), respectively. The genomes of these viruses have been characterized and compared to the three previously known piscine toti-like viruses, piscine myocarditis virus (PMCV) found in Atlantic salmon and the two from golden shiner, now named golden shiner toti-like virus 1 and 2 (GSTLV-1 and -2), and also to totiviruses and other toti-like viruses. We found that four piscine toti-like viruses had additional gene(s) in the 3′ end of the genome, and also clustered phylogenetically based on both capsid and RdRp-genes. This cluster constituted a distant branch in the *Totiviridae*, and we suggest this should be defined as a separate genus named *Pistolvirus,* to reflect this major cluster of piscine toti-like viruses. The remaining two piscine toti-like viruses differentiated from these by lacking any additional 3′ end genes and also by phylogenetical relation, but were both clustering with arthropod viruses in two different clusters.

## 1. Introduction

*Totiviridae* is a family of double-stranded RNA (dsRNA) viruses infecting uni-cellular organisms like fungi and protozoa. Five different genera have been recognized: The *Totivirus* and *Victorivirus* infecting yeast and fungi and the *Giardiavirus*, *Leishmaniavirus* and *Trichomonasvirus* infecting parasitic protozoa [1]. The viruses classified within the *Totiviridae* family are described as simple icosahedral non-enveloped viruses with genomes varying in size from 4.6 to 7.0 kb with two large and usually overlapping, open reading frames (ORFs). The 5′-proximal ORF1 encodes the capsid protein and the 3′-proximal ORF2 encodes an RNA dependent RNA polymerase (RdRp). The RdRp is virion-associated and usually expressed as a fusion protein following some copies of the capsid [1]. These viruses are associated with latent infections of their hosts and the virions remain intracellular and are transmitted during cell division, sporogenesis and cell fusion. *Giardiavirus* can also be released into extracellular environments and possesses the ability to infect hosts as purified particles [1].

In recent years, an increasing number of toti-like viruses have been discovered in more advanced hosts mainly due to next generation sequencing technologies (NGS). The majority of these have been found in arthropods, such as common fruit fly, mosquito, ants and ticks [2,3,4,5], the seawater habituating arthropods shrimp and horseshoe crab [5,6], as well as in roundworms and flatworms [5,7], bats (i.e., in feces, suggested to be due to insectivorous bats) [8], and the two piscine species, the Atlantic salmon (*Salmo salar*) and golden shiner (*Notemigonus crysoleucas*) [9,10,11]. Recently, metatranscriptomics studies of several fish species have shown new detection of short sequences from toti-like virus in samples from Atlantic salmon in Canada and in Australasian snapper (*Pagrus auratus*) [12,13], although whether the true host were the fish itself or a fungi species parasitizing on the fish, could not be concluded. Since totiviruses are associated with latent infections of their fungal or protozoan hosts, a similar course of infection could be assumed possible for several of the newer toti-like viruses, as these viruses have been reported from asymptomatic hosts, e.g., as reported for insect arthropods [2], worms [7], and the two piscine toti-like viruses found in golden shiner [10,11]. Disease has been proven to be caused by the infectious myonecrosis virus (IMNV), i.e., infectious myonecrosis (IMN) in shrimp, and piscine myocarditis virus (PMCV) causing cardiomyopathy syndrome (CMS) in salmon [9,14].

The arthropod and bat related viruses and one of two toti-like viruses found in the fish, golden shiner, all share the genomic characteristics described for *Totiviridae* but have a longer genome (6.8–8.2 kb). This is related to an ORF1 with additional sequences upstream to the capsid, encoding a putative RNA-binding protein and small protein of unknown function [2,3,4,6,8,10]. The toti-like virus found in Atlantic salmon, PMCV [9] and the other toti-like virus found in golden shiner, preliminary named PMCV-like virus [11], have more unique genomic characteristics. The size of ORF1 which is expected to encode the capsid, has a similar size as for the general totiviruses (i.e., does not include additional sequences upstream of the capsid as the other toti-like viruses). In contrast, these two viruses have additional sequences present as a single third ORF (ORF3) [9,11]. For PMCV, there have been several hypotheses on the function of the encoded protein of ORF3, such as being a part of the virion (a surface structure) or playing a role in enhancing or modulating the inflammation characterizing the disease following infection, due to partial homology with a chemokine superfamily motif [9,15]. In general, it is expected that the additional genomic sequences and expressed protein products in all the toti-like viruses is related to improving the ability to infect cells of a more advanced host than the standard totiviruses.

PMCV has previously been well characterized over its full-length genome [9,16], but a comparison of this virus with its related counterpart isolated from golden shiner, has been incomplete due to lack of full-length sequence. Here, we present for the first time the full-length genomic sequence of the PMCV-like virus found in Golden shiner from Minnesota, US, from now on referred to as golden shiner toti-like virus 1 (GSTLV-1). In addition, three novel piscine toti-like viruses are presented here: one from wild common carp (*Cyprinus carpio*) in Minnesota, USA; one from wild bluegill (*Lepomis macrochirus*), also Minnesota, USA; and one found in farmed lumpsucker (*Cyclopterus lumpus*) in Norway. The novel viruses are named common carp toti-like virus 1 (CCTLV-1), blue gill toti-like virus (BGTLV-1), and *Cyclopterus lumpus* toti-like virus (CLuTLV), respectively. The genomes of the novel viruses are characterized and compared to PMCV and updated full-length genome of GSTLV-1 and also to the second previous toti-like virus found in golden shiner (golden shiner totivirus) [10], which will from now on be referred to as golden shiner toti-like virus 2 (GSTLV-2).

The study combines detailed characterization and comparison of genomic characteristics of the novel and known piscine toti-like viruses with phylogenetic studies, also including other toti-like viruses and viruses of *Totiviridae*. The results increase the understanding of evolution of viruses related to *Totiviridae* and the expanding group of toti-like viruses and add knowledge to how these viruses adapt to infect in higher organisms. The study also highlights the emergence of novel toti-like viruses in different fish species that are economically and/or ecologically important throughout both the USA and Norway and global.

## 2. Materials and Methods

### 2.1. Origin of Samples and Virus

All analyses on PMCV were performed using the genomic sequence of the PMCV isolate AL V-708 (GenBank accession number HQ339954) [9].

New whole genome sequencing and genomic analyses on GSTLV-1 (previously called golden shiner PMCV-like virus) was performed on an individual pool sample of five fish confirmed to have high viral loads using PCR analyses in our previous work ([11] and Supplementary Text File S1).

Identification of viruses from common carp and bluegill, CCTLV-1, and BGTLV-1, originated from samples that belongs to a broad viral metagenomic study where dead fish were collected from natural mortality events in lakes distributed across the Upper Midwest region of the USA from June 2017 to October 2018. The methods for tissue collection and processing followed the recommended procedures in USFWS and AFS-FHS Blue Book (American Fisheries Society). Briefly, the brain, gill, spleen, and kidney tissues were aseptically collected from each fish and stored in Whirl-Pak^®^ sterilized sampling bags (Nasco, Thomas Scientific, Swedesboro, NJ, USA). The harvested tissues were stored at −80 °C until downstream nucleic acid extraction could be completed.

Characterization and genomic analyses of CLuTLV originates from samples of moribund lumpsucker fry collected from lumpsucker farms experiencing a sudden increase in mortality (up to 80%), in the mid-west of Norway during the spring of 2018. The lumpsucker fry collected midway through the mortality event showed no clinical signs or gross pathology. However, due to the small size of the fry (7–30 mm), whole fry was subjected to histological analysis performed at the commercial diagnostic laboratory at Pharmaq Analytiq (Oslo, Norway), and indicated increased presence of fluid in the intestine and gut. Lumpsucker fry samples (*n* = 3) were subjected to whole-genome sequencing (described below).

### 2.2. RNA Extraction, Library Preparation, and NGS

#### 2.2.1. GSTLV-1, CCTLV-1, and BGTLV-1

Samples for GSTLV-1 were subjected to RNA extraction using TRIzol LS Reagent (Invitrogen, Grand Island, NY, USA) followed by RNA purification using a QIAamp MinElute Virus Spin Kit (Qiagen, Germantown, MD, USA) as previously described [11]. For CCTLV-1 and BGTLV-1, stored frozen tissues were thawed to 25 °C and pooled according to tissue type sampled from each lake. A total number of 22 bluegill tissue pools and 83 common carp tissue pools were included in the study. Each tissue pool was homogenized using a stomacher in sterile, RNAase/DNAase-free 1× Phosphate Buffer Saline (PBS) solution in a 1:10 ratio (weight: volume) and centrifuged at 3200× *g* for 25 min at 4 °C. The supernatants were used for nucleic acid extraction and were first treated with TURBO™ DNAase and RNase (Ambion, Austin, TX, USA) for 1 h at 37 °C, to reduce host nucleic acid interference. Nucleic acids were extracted using QIAamp MinElute Virus Spin Kit (Qiagen, Germantown, MD, USA) by following manufacturer’s instruction. The extracted nucleic acids were submitted to the University of Minnesota Genomics Center for library preparation and sequencing.

The library preparation was completed using the dual-indexed Stranded Total RNA Pico Mammalian kit, according to the manufacturer’s instructions (Clontech/Takara Bio Company, Mountain View, CA, USA). Libraries were normalized according to the median fragment size measured by Tape Station 2.0 (Agilent, Santa Clara, CA, USA) and library concentration measured by Qubit. Sequencing was performed on the MiSeq platform (Illumina, San Diego, CA, USA) using a 150 bp pair end protocol (GSTLV-1) and HiSeq 2500 platform (Illumina) using a 2 × 125-bp paired end protocol (CCTLV-1 and BGTLV-1). After automated cluster generation, the genomic sequence reads (fastq) files were obtained for further analysis. The obtained Miseq sequence reads were analyzed using CLC Genomics Workbench, version 7.5 (Qiagen, Germantown, MD, USA) by following workflow of trimming adaptor sequences and sequence quality testing and de novo assembly. The HiSeq FASTQ files were analyzed using in-house bioinformatics pipeline for trimming to remove Illumina adapters using Trimmomatic, version 0.39; a flexible read trimming tool for Illumina NGS data; Usadellab: GitHub.com, [17] with a minimum quality score of 20. Then, bowtie2, version 2.4.4; a tool for aligning sequencing reads to long reference sequences; Ben Langmead: GitHub.com, [18] was used to remove host contamination and unmapped reads were used for assembly with SPAdes v.3.15.2; a genome assembler; Center for Algorithmic Biotechnology: GitHub.com, [19] with k-mer values of 21, 31, 41, 51, 61, and 71 and the—careful option. Extracted contigs were analyzed using BLASTx at NCBI to determine taxonomy. Contigs belonging to *Totiviridae* were subjected to ORFs prediction using Vgas; a tool for viral genome annotation; Centre for Informational Biology, University of Electronic Science and Technology of China: Chengdu, China [20] with default parameters.

#### 2.2.2. CLuTLV

Whole lumpsucker fry or tissue samples were homogenized in OTRK lysis buffer (Omega Bio-tek, Norcross, GA, USA) followed by centrifugation at 6000× *g* at 4 °C for 10 min. Homogenates were transferred to a Hamilton STAR robot (Hamilton Nordic AB, Kista, Sweden) and total RNA was extracted using MAG-BIND Total RNA kit (Omega Bio-tek, Norcross, GA, USA), after the manufacturer’s protocol. Concentration and purity of RNA were determined using a NanoDrop ND-1000 Spectrophotometer (NanoDrop Technologies Inc., Wilmington, DE, USA).

Total RNA (ca. 1000 ng/uL) were shipped to BaseClear (Leiden, Netherlands) for lllumina HiSeq strand-specific total RNA library preparation, followed by Illumina HiSeq 2500, 125 bp paired-end sequencing using the HiSeq SBS Kit v4 (Illumina, San Diego, CA, USA). FASTQ sequence files were generated using bcl2fastq2 v.2.18; software for demultiplexing and base calling, Illumina: San Diego, CA, USA. Initial quality assessment was based on data passing the Illumina Chastity filtering. Subsequently, reads containing PhiX control signal were removed and reads containing partial adapters were clipped up to a minimum read length of 50 bp. The second quality assessment was based on the remaining reads using the FASTQC quality control tool version 0.11.5. Raw Illumina reads were trimmed using “Trim Sequences” module in CLC Genomics Workbench version 11 (Qiagen), followed by assembly into contigs using the “de novo assembly” module, with the minimum contig size set to 120 bp. Open reading frames were predicted in the resulting contigs using Prodigal v.2.6.3; software for protein-coding gene prediction for archaea and bacteria; GNU Guix, GNU project, with a minimal sequence size set to 40 amino acids. The ORF sequences were then used to filter the contigs of less than 200 bases containing stop codons in all frames. The remaining contigs were aligned to a local copy of the NCBI nt database (downloaded February 2018) using BLAST v.2.6.0; software for finding regions of local similarity between sequences; NCBI, Bethesda, MD, USA. BLAST results were analyzed and contigs were split into the categories; Vertebrata, other eukaryotes, bacteria, virus and miscellaneous.

### 2.3. Sanger Sequencing of CLuTLV

To verify the ORFs in the CLuTLV genome sequence, sequencing primers were developed from the Illumina generated sequences to specify amplification of each ORF as a full product (described in patent file, see Section 5). The PCR reactions were set up using qScriptXLT One-Step RT-PCR kit (Quantabio, Beverly, MA, USA) according to the manufacturer‘s recommendations with 2.5 uL CLuTLV positive RNA (RT-qPCR CT value < 20) as the template. The following PCR program was used: initial denaturation step 94 °C, 2 min and subsequent 35 cycles of amplification (94 °C, 30 s; 55 °C, 30 s; 72 °C, 2 min). PCR products were run on a 1% agarose gel for verification of the product. Positive PCR product was cleaned using ExoCleanUp FAST (VWR Life Science, Radnor, PA, USA) and sequenced using a Big Dye Terminator v3.1 Cycle sequencing kit (Applied Biosystems^®^, Foster city, CA, USA) at the Sequencing Facility at the University of Bergen, Bergen, Norway.

### 2.4. Phylogeny

The study sequences were compared with totivirus sequences from GenBank. All sequences were aligned using MAFFT, v.7.450; a multiple sequence alignment program; [21] with scoring matrix BLOSUM62. Phylogenetic analysis was done using Geneious Prime 2020.2.2 (Geneious, Biomatters, San Diego, CA, USA) [22]. Maximum likelihood (ML) trees were generated from full genome alignment using RAxML-NG, version 1.0.2; a phylogenetic tree inference tool; Alexey Kozlov: GitHub.com [23]; ML trees were run with 100 bootstrap replications with a gamma distribution for rates over sites with protein model LG + G8 + F.

### 2.5. In Silico Sequence Analyses on Full Length Genomic Sequences and Protein Sequence Derivatives

Sequence analyses related to the genomes and encoded proteins were performed using the software CLC main workbench, version 6.9.2; CLCBio, Aarhus, Denmark and Geneious Prime, version 2020.2.2 (Geneious, Biomatters: San Diego, CA, USA. Initial homology searches were performed using Blastn and Blastp; NCBI: Bethesda, MD, USA, the latter also including searches for conserved domains in the proteins using DELTA-BLAST (Domain Enhanced Lookup Time Accelerated BLAST).

Prediction of H-type RNA pseudoknots in relation to potential ribosomal frameshift sites was performed using two programs, HPKNOTTER; Department of Biological Science and Technology, National Chiao Tung University: Hsinchu, Taiwan [24] and KnotInFrame, BiBiServ—the Bielefeld University Bioinformatics Server; Bielefeld University: Bielefeld, Germany [25,26], to verify some uncertainty in the results of such predictive programs. Mass and isoelectric point of encoded proteins are analyzed using Expasy compute pI/Mw tool; Swiss Institute of Bioinformatics: Lausanne, Switzerland [27]. Transmembrane regions of proteins were analyzed using TMpred; Swiss Institute of Bioinformatics: Lausanne, Switzerland [28,29] and signal peptides were discriminated from these using SignalP, v.5.0; Center for Biological Sequence Analysis, Denmark technical university: Lyngby, Denmark [30,31]. PSORT, version II; GenScript: Leiden, Netherlands [32] was also used to confirm the signal sequence and transmembrane regions. Conserved domains were also confirmed using the protein structure homology-modelling server SWISS-MODEL; Swiss Institute of Bioinformatics: Basel, Switzerland [33]. Only predictions scored above levels for significant quality, as given in each software, are included as results. A few exceptions to this occur as it could have relevance in a larger context. These exceptions are specified as low score predictions.

## 3. Results

### 3.1. Full-Length Genomic Sequence of Golden Shiner Toti-Like Virus 1 (GSTLV-1)

GSTLV-1 nearly complete genome with complete ORFs was assembled from one individual sample of the five pooled fish used for previous sequencing [11]. The sequence is identical with the sequence reported previously, but also adds more information to 5′ and 3′ ends of the genome. The genome consists of 6761 nts and is described more below.

### 3.2. Novel Toti-Like Viruses Detected in Common Carp and Bluegill

Toti-like virus sequences were detected in common carp and bluegill fish in individuals collected from natural mortality events in lakes in Minnesota (Supplementary Text File S1). The viruses are named as common carp toti-like virus (CCTLV-1) and bluegill toti-like virus (BGTLV-1).

The nearly complete genome of CCTLV-1 was assembled from sequences of pooled kidney samples from Lake Ballantyne, while partial sequences were detected in an additional 13 pooled tissue samples from various lakes (Supplementary Text File S1). The nearly complete genome of BGTLV-1 was assembled from pooled spleen tissue from Lake Waconia and partial sequences were also detected in pooled gill tissue from the same lake (Supplementary Text File S1).

### 3.3. Novel Toti-Like Virus Detected in Lumpsucker

Sequencing of samples from moribund lumpsucker fry from a mortality event in a lumpsucker farm, revealed a virus with similarities to the toti-like viruses PMCV and GSTLV-1.

Although original samples for detection of CLuTLV originated in moribund fry and subsequent PCR screening for this virus showed repeated detection in samples including milt and eggs from broodstock, lumpsucker fry, and different tissue samples such as gills, liver, spleen and kidney tissues from adult fish (Supplementary Text File S1), no clinical signs have been associated with CLuTLV. The moribund lumpsucker fry from the outbreak in spring 2018 where CLuTLV was originally detected, was affected with a second virus that later on has been linked to the clinical signs and sudden mortality (Sandlund/Nylund, personal observations from field material). The second virus shared similarities with viruses of the Coronaviridae and will not be further discussed here.

Lumpfish is used as cohabitants with Atlantic salmon as they feed on sea lice parasitizing on the salmon skin. To study a possible transfer of CLuTLV to salmon, screening has been performed in farmed Atlantic salmon in several production stages and all were negative for the virus (Supplementary Text File S1). Ballan wrasse (*Labrus bergylta*) may be similarly used as cleaner fish for sea lice on the salmon and in a small screening study on ballan wrasse, low levels of CLuTLV was detected in the kidney of one ballan wrasse (*Labrus bergylta*) sampled in May 2018 (Supplementary Text File S1).

### 3.4. Piscine Toti-Like Viruses Share Major Genomic Characteristics

**Figure 1:**
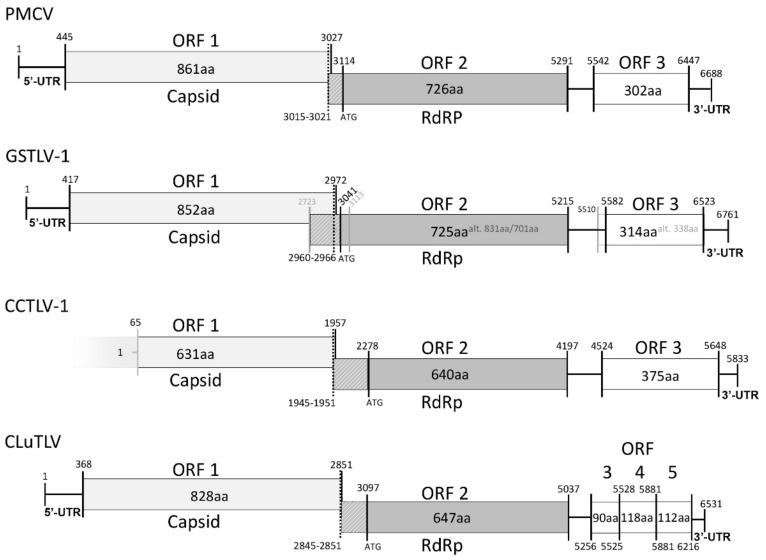
Schematic overview of genomes of piscine toti-like viruses including additional 3′ end open reading frame(s) (ORF). Nucleotide (nt) numbering given as first nt of start codon and last nt of last translated codon. ORF2 is given including putative start codon(s) and resulting number of amino acids (aa) encoded, but for PMCV, GSTLV-1, and CCTLV-1, position for a characteristic slippery site which can result in translation of ORF1 and ORF2 as a fusion protein is also shown by dotted line with genomic position of site. A putative slippery site is also indicated similarly for CLuTLV. For GSTLV-1 an alternative start codon for ORF2 and ORF3 is indicated in grey lines. All sizes of boxes defining ORFs and lines defining untranslated regions (UTRs) are in correct ratio and each genome is centered around ORF2 in all to ease visual size comparison. Genomic information is expected to be incomplete in 5′ end of CCTLV-1.

The new full-length genomic sequences of GSTLV-1 and the three novel viruses were analyzed according to the length of total genome, number and characteristics of ORFs found, and untranslated regions (UTRs) and characteristic sites and patterns and compared to those previously found for PMCV (Figure 1 and Table 1). For the viruses from common carp and bluegill, near full-length genomic sequences were obtained and theses viruses were similarly compared, as far as possible.

**Figure 2:**
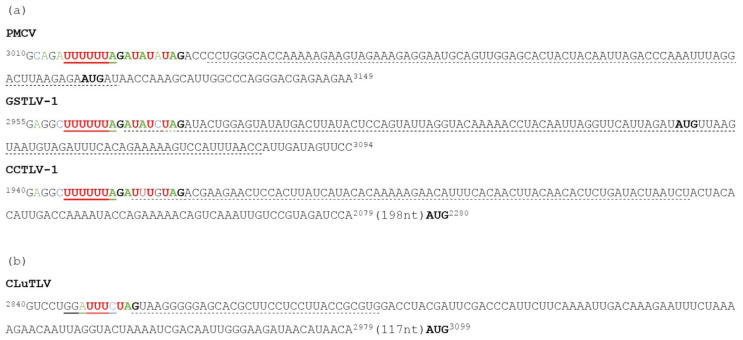
Characteristics of sequences related to ribosomal frameshift for translation of ORF2 as a C-terminal fusion protein with the ORF1 encoded protein. Nucleotides in 3’ end of ORF1 are color coded. (**a**) PMCV, GSTLV-1 and CCTLV-1. Preserved nucleotides are shown in bold and heptameric nucleotide slippery sequences are underlined. (**b**) Suggested slippery sequence in CLuTLV genome underlined. For all, ORF1 stop codon (UAG) and first possible start codon (AUG) of ORF2 if expressed non-fused to ORF1, are shown in bold. Sequences predicted to result in pseudoknots by dotted underlining (KnotInFrame predicted: PMCV, GSTLV-1 and CCTLV-1 in (**a**), HPknotter predicted: CLuTLV in (**b**). Numbers on sequences refer to position in full length genome.

The genome of GSTLV-1 has been partly described previously [11], but the new sequencing resulted in additional nucleotides in both 5′ and 3′ (GenBank accession MW888449, Figure 1 and Table 1) and subsequent analyses revealed new genomic characteristics. For this virus a new in-frame start codon was revealed for ORF1 with a new resulting length of 2556 nt. The predicted ORF2 still shows overlap of ORF1 in a -1 frameshift relative to ORF1 (Figure 1). A slippery site was found in the 3′ end of ORF1. This is defined as the heptameric nucleotide sequence N NNW WWH, where the incoming reading frame is indicated by spacing and both N and W must be stretches of three identical nucleotides [34,35]. The sequence was identical to the U UUU UUA site found in PMCV and was followed by six nucleotides, of which five were also identical to PMCV, before the UAG stop codon of ORF1 [9] (Figure 2). For both PMCV and GSTLV-1 we found regions of the RNA in close proximity downstream of the slippery sites predicted to result in h-pseudoknots (Figure 2). This indicates that both of these viruses use -1 ribosomal frameshifting with the resulting translation of ORF2 as a C-terminal fusion protein with the ORF1 encoded protein.

The more complete genomic sequence of GSTLV-1 also revealed a full-length ORF3. The prediction of ORFs suggests an ORF3 with 1014 nts encoding a protein of 338 amino acids (aa). A second ATG codon is found 72 nts downstream of the predicted ATG start codon, which would result in an encoded protein sized 314 aa (Figure 1 and Table 1). Analyses of the encoded 338 and 314 aa proteins, in comparison to PMCV ORF3, indicate that the latter ATG codon resulting in an ORF3 sized 945 nts encoding the 314 aa variant is used (see below).

For CCTLV-1 found in common carp, a genomic sequence of 5833 nts has been characterized, also including three ORFs (GenBank accession MW893686, Figure 1 and Table 1). Compared with the other piscine toti-like viruses the 5′ UTR is very short (64 nt) and ORF1 is also shorter (1893 nt) (Table 1); however, it is expected that the sequence in the 5′ end is not complete. A putative ORF2 starts 317 nt downstream of the ORF1 stop codon, in reading frame -1 relative to ORF1. Similar to PMCV and GSTLV-1 a slippery site was found in the 3′ end of ORF1. The sequence was identical to the U UUU UUA site found in both PMCV and GSTLV-1 and was followed by six nucleotides, of which four were identical to both PMCV and GSTLV-1, before the UAG stop codon of ORF1 (Figure 2). Furthermore, also here, h-pseudoknots were predicted in regions of the RNA in close proximity downstream of the slippery sites, supporting -1 ribosomal frameshifting and resulting in ORF1 and ORF2 expressed as a fusion protein. A putative full-length ORF3 is found for this virus, with size 1125 nts.

The complete genome of CLuTLV consists of 6531 bp including five ORFs (GenBank accession number MW811138, Figure 1 and Table 1). The putative ORF1 has a length of 2484 nts. Furthermore, for this virus, the putative ORF2 is present downstream of ORF1 in reading frame -1 relative to ORF1. A slippery site defined as the classical nucleotide sequence N NNW WWH promoting a shift, as found for PMCV, GSTLV-1 and the CCTLV-1, was not present in CLuTLV. Still, the last nts preceding the stop codon of ORF1, GGAUUU C (Figure 2), is similar to GGAUUU A/U described in the Frameshift Signal Database [34], except for of the last nt. Moreover, sequences predicted to result in pseudoknots were found downstream of this putative site, indicating that co-translation of ORF1 and ORF2 as a fusion protein is possible after a -1 ribosomal frameshift. A UTR of 215 nts is found downstream of ORF2 followed by three small ORFs (ORF3, 4 and 5). These ORFs are overlapping each other by only a few nts (Figure 1) and in -1 frameshift for ORF4 relative to ORF3 and also ORF5 relative to ORF4.

For the BGTLV-1, a genomic sequence of 5307 nts was characterized, including two ORFs (GenBank accession MW893687, Table 1). Similar to CCTLV-1, the 5′ and 3 ′UTR are shorter than found for the other three piscine toti-like viruses and it is expected that sequencing of the ends of this genome is not complete. Any additional ORFs, such as ORF3, were not found. This is comparable to the piscine toti-like virus GSTLV-2, found in Golden shiner [10], but could also be due to an incomplete sequencing result excluding any additional ORFs. The predicted ORF1 is 2673 nts, encoding a protein of similar size as the ORF1 encoded proteins of the other piscine toti-like viruses (Table 1), with the exclusion of GSTLV-2, for which ORF1 is of larger size and encodes a polypeptide. It is therefore unknown if the predicted ORF1 is of full-length and comparable to ORF1 of PMCV, GSTLV-1 and CLuTLV, or if it is not complete at the 5′ end and should be longer and comparable to GSLTV-2. A putative ORF2 seems to overlap ORF1 by 10 nts in a -1 frameshift relative to ORF1, but no known -1 ribosomal frameshift site has been found. ORF2 is 2355nts long (Table 1).

### 3.5. Characteristics of the Putative Capsid Encoded by ORF1 and RdRp Encoded by ORF2

The ORF1 of the CCTLV-1, CLuTLV and BGTLV-1, all putatively encode the capsid of the virus as previously predicted for PMCV and GSTLV-1, and other toti-like viruses [2,3,4,6,8,10]. Blast homology searches with the predicted amino acids sequence of the CCTLV-1 and CLuTLV ORF1s in GenBank showed a match with high significance (based on Blast E-value) to both PMCV and GSTLV-1 ORF1. ORF1 encoded protein of the BGTLV-1, showed the highest homology to Hypothetical protein 1 (i.e., ORF1 encoded homologue) from Beihai toti-like virus 4 strain HOU154008 detected in horseshoe crab (GenBank YP_009336712.1, [5]), and also significantly similar to PMCV, GSTLV-1, and CLuTLV. Similar to the PMCV and GSTLV-1 ORF1 encoded proteins, neither 2A-like motifs (GDVESNPGP and GDVEENPGP) nor dsRNA binding motif (DSRM) [14,36] were found in the protein encoded by ORF1 of any of the novel piscine toti-like viruses. Although as discussed above, the 5′ genomic end of BGTLV-1 is anticipated to be incomplete, which might result in missing amino acids at the N-terminal end of where these motifs typically would be positioned. This might also apply to the CCTLV-1 ORF1 encoded protein. No conserved domains are found in any of ORF1 protein sequences.

The amino acid sequence of CCTLV-1 and CLuTLV ORF2s match with significant similarity to both PMCV and GSTLV-1. For the BGTLV-1 ORF2 protein, a significant match is only found to a smaller region of the GSTLV-1 ORF2 encoded protein. Additionally, as previously presented for PMCV and GSTLV-1, ORF2 protein of CCTLV-1 and CLuTLV show sequence similarities to the RdRp part of the gag-pol fusion protein (i.e., capsid-RdRp fusion protein) of Giardia lamblia virus. An even higher similarity is found for the BGTLV-1 ORF2 protein of the presented viruses to several briefly characterized toti-like viruses (e.g., hypothetical protein 3 from the Hubei toti-like virus 17 found in ticks and hypothetical protein 2 from Beihai toti-like virus 4 found in horseshoe crab, [5], both genomic position homologues to RdRps of these viruses).

**Figure 3:**
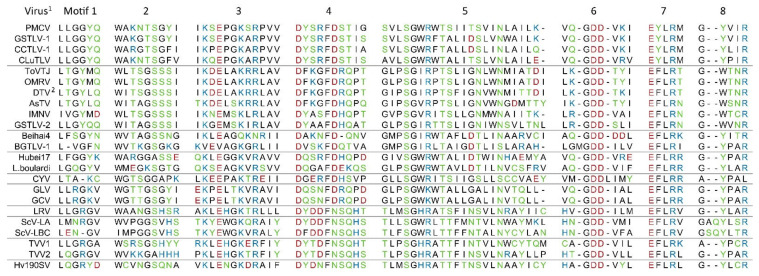
Alignment of the piscine toti-like virus RNA-dependent RNA polymerases (RdRp) motif 1–8 with RdRp motif equivalents of selected members of the family *Totiviridae* and toti-like viruses. Order and grouping are given according to clusters resulting from phylogenetic analyses (Figure 6b). Amino acids are color coded on polarity; Black non-polar, green polar uncharged, red polar acidic, blue polar basic. Definition of motif length is based on a synergy of previously used by Poimala et al., 2020, Poulos et al., 2006 and original paper describing the motifs of viruses registered in *Totiviridae* in Bruenn et al., 1993 [14,37,39]. Accession numbers to sequences used can be found in Supplementary Table S1. ^1^ Virus abbreviations: Piscine myocarditis virus (PMCV), golden shiner toti-like virus 1 (GSTLV-1), common carp toti-like virus 1 (CCTLV-1), *Cyclopterus lumpus* toti-like virus (CLuTLV), Tianjin totivirus (ToVTJ), Omono river virus (OMRV), drosophila totivirus (DTV), *Armigeres subalbatus* totivirus (AsTV), infectious myonecrosis virus (IMNV), golden shiner toti-like virus 2 (GSTLV-2), Beihai toti-like virus 4 (Beihai4), bluegill toti-like virus 1 (BGTLV-1), Hubei toti-like virus 17 (Hubei17), *Leptopilina boulardi* toti-like virus (L. boulardi), *Camponotus yamaokai* virus (CYV), *Giardia lamblia* virus (GLV), *Giardia canis* virus (GCV), Leishmania RNA virus (LRV), *Saccharomyces cerevisiae* virus L-A (ScV-LA), *Saccharomyces cerevisiae* virus L-BC (La) (ScV-LBC), *Trichomonas vaginalis* virus 1 (TVV1), *Trichomonas vaginalis* virus 2 (TVV2), and *Helminthosporium victoriae* 190S virus (Hv190SV). ^2^ Motif 1 is found outside defined ORF2 in part included between ORF1 and ORF2 if expressed as a fused protein.

The similarity to the RdRp of registered totiviruses strongly indicates a similar function of ORF2 encoded protein in the piscine toti-like viruses. This is also strengthened by findings of a conserved domain, general to the reverse transcriptase like superfamily (RT_like family, cl02808) and a viral RdRp domain (RDRP_4, pfam02123) for all. Furthermore, eight motifs characteristic of totivirus RdRp [14,37] were identified in the RdRps of all the piscine viruses (Figure 3), also including the universal RNA polymerase GDD [38] in motif 6.

The identity of the motifs is high among the four piscine viruses PMCV, GSTLV-1, CLuTLV, and CCTLV-1 and show moderate identity to other toti-like viruses and the totiviruses, including the two other piscine viruses, GSTLV-2 and the novel BGTLV-1 (Figure 3). These protein comparisons and analyses have been made based on the encoded amino acids of predicted ORF2 from the start codon to the last translated codon. A sequence equal to a -1 ribosomal frameshift site is detected in all the piscine viruses (putative sites found in CLuTLV and BGTLV-1), as described above. If an RdRp is expressed fused to the C-terminal end of the capsid from ORF1 and the size of the RdRp gene is predicted from the site where the ribosomal frameshift happens, the resulting sizes of the RdRp gene are comparable for all the piscine viruses presented (Table 1b).

### 3.6. Characteristics of ORF3 Encoded Protein in PMCV, GSTLV-1, and CCTLV-1

**Figure 4:**
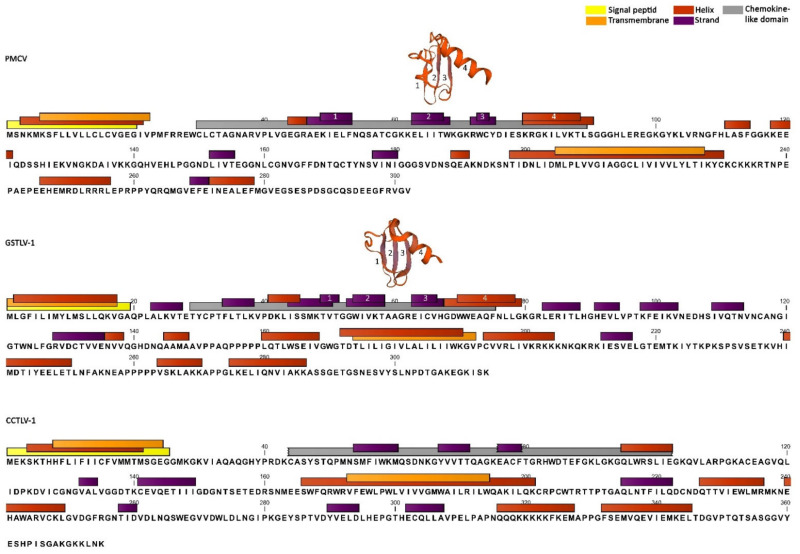
Overview of predicted domains for signal peptide, chemokine-like domain, transmembrane region and predicted α-helices and β-strands for PMCV ORF3, GSTLV-1 ORF3 (314aa), and CCTLV-1 ORF3. Predicted 3D models of chemokine-like domain are included for PMCV and GSTLV-1 close to the domain in the sequence and the three strands and one helix are marked by numbering corresponding between sequence and 3D model. The models are predicted against platelet factor 4, i.e., CXC chemokine 4 and CC chemokine 2, respectively. One predicted β-strand with low quality score is included for CCTLV-1, as it could have relevance for a tentative chemokine-like motif. This strand and also tentative chemokine-like motif are marked by tagged ends of bars to show high uncertainty of predictions/length. Predictions of hydrophobic regions are not included separately using colored bars but are consistent with signal peptide/transmembrane predictions.

The encoded protein of PMCV ORF3 has previously been described to have partial homology to CXC chemokine 11, including a chemokine superfamily motif, in the N-terminal part of the protein [9]. A signal peptide was now found upstream of this, i.e., represented by the first 20 aa at the N-terminal end (Figure 4). Transmembrane predictions and predictions of regions of high hydrophobicity confirmed these 20 aa to include a high concentration of hydrophobic amino acids and to have a putative relation to membranes, a typical characteristic of signal peptides as they direct proteins to either location in a membrane or to be secreted to extracellular environments from a membrane. A region of 23 aa was also found in the second half of the protein for which a high score was found for transmembrane characteristics, coincident with a high concentration of hydrophobic amino acids (Figure 4, PMCV).

Recent searches for conserved domains and structure homology modelling of PMCV ORF3 showed, as previously described, similarity to several chemokines of the CXC subfamily, including highest similarity to interleukin 8 (i.e., CXC chemokine 8) from NCBI (e-value 3e^−14^–1e^−18^). The domain includes the characteristic two N-terminal cysteines, separated by one amino acid for CXC chemokines, followed by three stretches of β-strands in all chemokines comprising a sheet structure and a α-helix in the C-terminal end of the motif, which were all confirmed by in silico prediction of secondary structures and protein structure homology-modelling (Figure 4).

The genome of GSTLV-1 has two possible start codons for ORF3 resulting in either an encoded protein length of 338 or 314 aa (Figure 1). The 314 aa variant is closer in length to the PMCV ORF3-encoded parallel with 302 aa. Homology analyses of the 314 aa variant of GSTLV-1 ORF3 resulted in only 19.06% identity of the amino acid sequence to PMCV ORF3, also including several gaps varying in length from one amino acid to several tens. Still, the encoded protein shares characteristics in presence and positions of a signal peptide, a chemokine-like domain and a stretch of hydrophobic amino acids/predicted transmembrane domain (Figure 4). If the additional amino acids are included in the N-terminal end with a resulting length of 338 aa, an N-terminal signal peptide is not found. The chemokine-like domain from GSTLV-1 ORF3, showed similarity by NCBI conserved domain searches to the CC motif chemokine-like 3 from brown trout (*Salmo trutta*) only, with a resulting weak e-value of only 0.013. Only one of the two characteristic adjacent cysteines near the N-terminal end of CC motif chemokines was found, but secondary structure predictions showed the characteristic three β-strands and subsequent α-helix. Protein structure homology-modelling predicted highest homology to CC motif chemokine 2 and also confirmed the positions of predicted secondary structures (Figure 4).

The ORF3 found in the genome of the CCTLV-1 encodes a protein of 375 aa, remarkably longer than the ORF3-protein of PMCV and the 314 aa variant of GSTLV-1. This protein shares the highest similarity (20.60%) with the GSTLV-1 compared to the PMCV ORF3 (14.49%), although comparative alignments of all three viruses include several gaps also here of varying sizes. Although there was low identity among the three ORF3-encoded proteins, the common carp variant was found with the signal peptide in the N-terminal end and the stretch of hydrophobic amino acids/transmembrane characteristics in the second half of the protein (Figure 4). No parts of the CCTLV-1 ORF3 encoded protein showed homology to a chemokine-like domain in either NCBI conserved domain searches or searches for protein structures for homology-modelling. Still, secondary structure predictions showed the characteristic three β-strands followed by an α-helix of chemokines in the N-terminal end subsequent to the signal peptide, similar to the chemokine-like domains of PMCV and GSTLV-1 ORF3, which indicate a similar characteristic of the CCTLV-1 too (Figure 4).

Secondary structure predictions also confirmed helix structure in relation to the N-terminal signal peptide/high hydrophobic region in the N-terminal end for ORF3 encoded protein in all three viruses and also similarly, a helix was predicted for the stretch of amino acids with high hydrophobicity in the second half of all three protein variants (Figure 4).

### 3.7. Characteristics of CLuTLV ORF 3, 4, and 5

**Figure 5:**
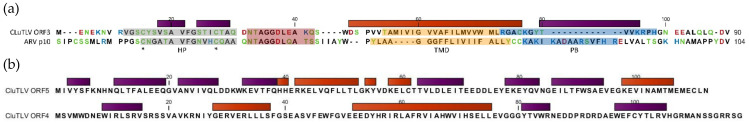
Characteristics of CLuTLV ORF3, 4 and 5 encoded proteins. (**a**) Alignment of ORF3 encoded protein against *avian orthoreovirus* (ARV) p10 (GenBank accession no. AKH03114). Conserved domain in red shade. Predicted transmembrane domains (TMD) shown in orange shade, other hydrophobic path (HP) in grey shade. Asterisks mark conserved cysteines. Polybasic motif (PB) in blue shade. Amino acids are color coded on polarity; Black non-polar, green polar uncharged, red polar acidic, blue polar basic. Predicted α-helices (red box) and β-strands (purple box) are included for CLuTLV. (**b**) Secondary structure predictions for CLuTLV ORF4 (i) and ORF5 (ii) encoded proteins given as described for ORF3 in (**a**) and other viruses in Figure 4.

Neither of the proteins encoded by the CLuTLV ORF 3, 4 and 5 share any significant homology with ORF3 of the three other viruses described here. NCBI Blastx searches revealed that the CLuTLV ORF 3 amino acid sequence shares 40% identity (with 67% coverage, i.e., excluding N-terminal and C-terminal ends) to the non-structural membrane protein p10 from *avian orthoreovirus*, a Fusion-Associated Small Transmembrane (FAST) protein responsible for cell membrane fusogenic properties [40]. The identity found relates specifically to a stretch of eight conserved amino acids in ORF3 from a total of 11 aa conserved between p10 of various *avian orthoreoviruses* (Figure 5a). In addition, two hydrophobic domains are found in CLuTLV ORF3 encoded protein, consistent with a hydrophobic path (HP) and transmembrane domain (TMD), for which the latter was also consistent with predictions of α-helixes, all found in p10 proteins (Figure 5a). The HP also included conserved cysteines and the TMD was followed by a motif consisting of several basic amino acids (polybasic, PB), both characteristics found in p10. Similar to p10, no signal peptide is found in the N-terminal end of the encoded protein.

For the CLuTLV ORF 4 and ORF 5 encoded proteins, significant homology is not found for any other known proteins (NCBI blastx) and no predicted signal peptide or hydrophobic/transmembrane domains are found. The prediction of β-strands and α-helixes did not show any recognizable pattern (Figure 5b).

### 3.8. Phylogenetic Relation between Piscine Toti-Like Viruses and to Other Toti-Like Viruses and Totiviridae

**Figure 6:**
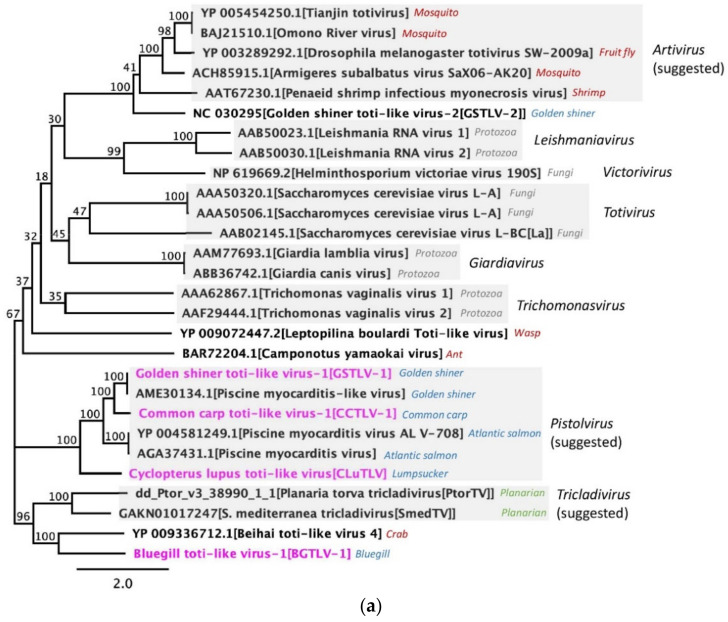

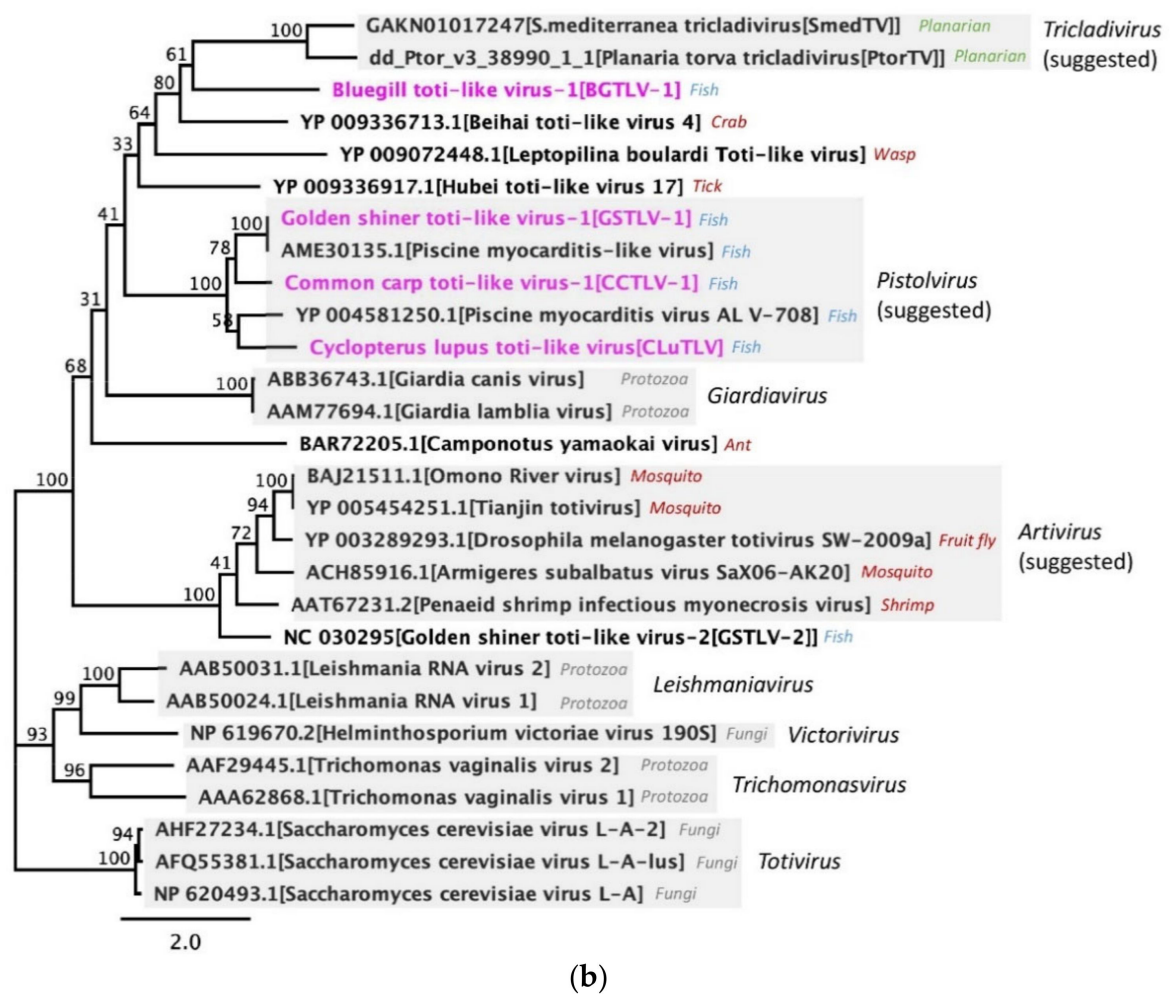
Phylogenetic relation of the six piscine toti-like viruses to members of the family *Totiviridae* and non-registered toti-like viruses, with annotations of registered and suggested new genera. The novel piscine toti-like viruses are highlighted in pink. The remaining viruses are referred to by GenBank accession numbers and virus name. Common host specie names are included for reference (uni-cellular organisms in grey, arthropods in red, planarians in green and fish in blue). Suggestions of new genera are according to Dantas et al., 2016 [41], Burrows et al., 2020 [7] and present work. The tree was constructed based on the complete capsid protein (ORF1) in (**a**) and complete RdRp protein (ORF2) in (**b**).

Phylogeny was used to establish and study the relationship between the novel and previously discovered piscine toti-like viruses, also including comparison with other toti-like viruses and virus representatives from the five established genera of *Totiviridae* (Figure 6). As seen previously, PMCV from Atlantic salmon in Norway and GSTLV-1 from golden shiner in the USA, constitute a distinct branch with clear distance to both registered totiviruses as well as arthropod and other recently described toti-like viruses [7,9,11]. The novel American CCTLV-1 from common carp and Norwegian CLuTLV from lumpsucker clustered with PMCV and GSTLV-1 in both capsid/ORF1 protein and RdRp/ORF2 protein based phylogeny (Figure 6), with 67.66–76.44% similarity based on the ORF2 proteins (Supplementary Tables S2 and S3). Although detected in piscine species, GSTLV-2 from golden shiner and BGTLV-1 from bluegill, both from USA, did not cluster with the other four piscine toti-like viruses. GSTLV-2 clustered with the IMNV sequences and other arthropod toti-like viruses, as seen previously [10], including 69.81% similarity to IMNV RdRp/ORF2 protein. The BGTLV-1 sequence is highly divergent with the highest similarity (51.68%) to Beihai-like virus 4 from horseshoe crab, based on ORF2 protein (Figure 6b and Supplementary Table S3). Similar clustering pattern was observed for all, based on ORF1 proteins, but with lower percent similarity.

## 4. Discussion

Viruses sharing similarities with the five genera of *Totiviridae* are increasing in number, complexity, and host species diversity. Here, we describe and compare the most divergent ones relative to the registered totiviruses, in both genomic characteristics and host phylogenetic order. Included among these are three novel viruses detected in the wild fish species common carp and bluegill and in farmed lumpsucker, here described for the first time. These are compared to PMCV infecting farmed Atlantic salmon [9] and its counterpart detected in the baitfish golden shiner, GSTLV-1 [11], for which a complete genomic sequence is now described, and also partly to GSTLV-2 found previously in golden shiner [10]. A characteristic feature of all toti-like viruses discovered in the more advanced hosts, compared to the uni-cellular host of the registered viruses of *Totiviridae*, is usually a longer genome. The genomic length of registered viruses is between 4.5 and 6.7 kb, while the toti-like viruses range between 5.7 kb for the *Camponotus yamaokai* virus (CYV) detected in ants [42] and 8.2 kb of the IMNV [6]. The additional length of these more novel viruses, of which the majority are found in arthropods, is generally related to additional sequences preceding the capsid coding sequences of ORF1 [2,3,4,6,8]. The piscine toti-like virus GSTLV-2 and putatively the novel BGTLV-1 presented here, have characteristics similar to the arthropod viruses [10]. PMCV and GSTLV-1, and the novel CCTLV-1 and CLuTLV, are all unique in having additional separate ORFs downstream of the traditional ORFs related to capsid and RdRp proteins.

For both PMCV, GSTLV-1 and CCTLV-1, a slippery site characteristic of a -1 ribosomal frameshift [34,35] is identified in the 3′ end of ORF1. In addition, we found that both this site and five of the six subsequent nts before the stop codon were preserved between the PMCV and GSTLV-1 and that CCTLV-1 had four of these nts preserved. Although no functional studies of the slippery sites have been performed for the two viruses, it fits well with -1 ribosomal frameshifting described for the genera *Giardiavirus*, *Leishmaniavirus*, and *Totivirus* of *Totiviridae* [1]. Similar characteristics are described in the genomes of the more novel and advanced toti-like viruses [2,4,8,10,36,42], including Omono river virus (OMRV) and Tianjin totivirus (ToVTJ), which share exactly the same nts in the slippery site as PMCV, GSTLV-1 and CCTLV-1. Since the presence of a slippery site described as the heptameric nucleotide sequence N NNW WWH [34,35] is well conserved between these viruses, it is more unexpected that we did not identify characteristics of a similar site in CLuTLV. On the contrary, a putative GGA UUU C shift site was identified, which tentatively could result in the ORF1/ORF2 polyprotein. The almost identical shift site GGA UUU U/A, differing only in the last nucleotide, is found the capsid protein of *Saccharomyces cerevisiae* virus L1 and La [43]. Recently, the shorter but identical UUU_C was also proposed as a slippery site for fusion of ORF1 and 2 in +1 or -2 frameshift, in the flatworm toti-like viruses [7], all supporting our finding that the frameshift site identified in CLuTLV is authentic. Although the use of -1 ribosomal frameshift seems to be a common characteristic for toti- and toti-like viruses, exceptions occur, as mentioned for the flatworm viruses. Moreover, *Trichomonasvirus* expresses capsid and RdRP as a fusion protein but might use either -1 or a +1 ribosomal frameshifting [44]. *Victorivirus* has the ORF2 in -1 frame relative to ORF1, but the RdRp encoded by ORF2 is expressed as a separate, non-fused minor protein [1]. The ORF2 of CLuTLV has a putative ATG start codon 242 nts downstream of ORF1 that could function as a start codon in use if it was expressed separately. Similar to the other piscine toti-like viruses, ATG codons that may function as start codons are present and define a separate ORF2 either overlapping with ORF1 or positioned a short distance downstream of ORF1. Still, the fact that the majority of toti and toti-like viruses seem to have either confirmed -1 ribosomal frameshift resulting in capsid-RdRp fused protein or typical genetical characteristics for this, indicates that the piscine viruses also express a capsid-RdRp fused protein. This is supported by more similar sizes of the RdRp part expressed after -1 frameshift, than if expressed as single ORFs.

A common characteristic among the recently discovered toti-like viruses infecting arthropods and fish is that they are known or presumed to transmit extracellularly. Specific genomic characteristics are suggested to be related to this, as all have extra protein-coding sequences that may encompass all or some of their cell entry or exit machineries [9,14,41]. The piscine viruses PMCV, GSTLV-1, CCTLV-1, and CLuTLV are unique among these viruses, as they are the only ones to have the extra protein-coding sequences as separate gene(s) located at the 3′ end, downstream of the totivirus-like organization of ORF1/capsid and ORF2/RdRp. In comparison, all arthropod viruses have their extra sequences located in the 5′ end of the genome and the same ORF as the capsid, i.e., translated as a polyprotein [41], a characteristic that is also seen in the piscine virus GSTLV-2. The BGTLV-1 clustered by phylogeny with the Beihai toti-like virus 4 found in horseshoe crab and could also be expected to express a similar polyprotein encoded ORF1, but the genomic sequences of the 5′ and 3′ ends are incomplete and therefore not possible to conclude.

The role of these additional protein(s) remains to be characterized, although extensive research is ongoing related to PMCV ORF3 (AB Mikalsen) and GSTLV-1 ORF3 (AB Mikalsen in collaboration with SK Mor). Previous hypotheses describing that PMCV ORF3 encodes a non-structural protein which is related to these virus’s ability to infect and/or is released from cells of a more advanced host, is still valid. The in silico predictions indicating a signal peptide followed by a chemokine-like domain in ORF3 of PMCV, GSTLV-1, and tentatively CCTLV-1, is interesting. Signal peptides are short peptides in the N-terminal end of proteins that direct these proteins to be secreted or to end at a transmembrane location if a second hydrophobic stretch of amino acids anchors the protein in a membrane. A secretory pathway fits well with the following chemokine-like domain, although the stretch of hydrophobic amino acids in the second half of these proteins, predicted to have an α-helix secondary structure, would fit well with being a transmembrane anchor. Preliminary results from expression studies of PMCV ORF3 in cell culture indicate that the ORF3 product is post-translationally processed into smaller parts, of which the N-terminal end with a size consistent with the chemokine-like domain, is found to be secreted to the supernatant after expression in vitro [45]. This indicates that the chemokine-like domains of the ORF3 encoded proteins in these viruses might be present upon infection as secretory small proteins. More documentation is ongoing and should also indicate if the similarity to chemokines indicates a role in modulation of the immune response or if binding to a chemokine receptor facilitates viral entry into cells.

The additional genomic sequences downstream of the traditional ORF1 and ORF2 is a bit different for CLuTLV with three very small ORFs replacing the ORF3 seen in PMCV and GSTLV-1. The three ORFs are non-overlapping in coding sequences, but close enough to have a few nts related to stop/start codons in common. The CLuTLV ORF3 shares identical amino acids with the non-structural protein p10 from *avian orthoreovirus*, a FAST protein of fusogenic orthoreoviruses, which are responsible for cell–cell fusion and syncytium formation [40]. Such FAST proteins contain a membrane spanning domain and a membrane-proximal cluster of basic amino acids and are generally highly basic with pI > 8.5 [46]. For the CLuTLV ORF3 encoded protein, a polybasic motif can be found following the C-terminal end of the predicted transmembrane domain, but the full protein is generally acidic (Figure 5 and Table 1). Still, since also a hydrophobic path including conserved cysteines and eight of a total of 11 amino acids conserved between p10 of various avian orthoreoviruses are found, it is reasonable to suggest that this might be a protein with similar characteristics as FAST proteins. They play no role in virus entry but are transcribed late in the infection cycle and trafficked to the plasma membrane to induce cell–cell fusion with neighboring uninfected cells. Such syncytiogenesis results in cell–cell transmission of the virus and is followed by apoptotic disruption resulting in virus progeny release. This could be a mechanism used by toti-like viruses like CluTLV as well. How CLuTLV have gained this putative FAST-protein encoding gene cannot be concluded. A recombination or reassortment event with an orthoreovirus could be suggested. Still, the closest orthoreovirus candidate (by host species) would be piscine orthoreovirus (PRV) which has ubiquitous presence in Norwegian Atlantic salmon aquaculture [47], where lumpsucker might be cohabiting with the salmon. The p10 analogue in PRV is p13, but although it shares similarities in being a non-structural membrane protein, this protein is not found to have fusogenic properties. Instead, it is a cytosolic, integral membrane protein which induces cytotoxicity in cells upon expression in vitro [48]. Moreover, no significant homology was found between the CLuTLV ORF3 and PRV p13 protein. Such a FAST protein encoding gene may also be a conserved domain from an ancient common ancestor virus, but this will need to be addressed in future studies.

Phylogenetic studies show that piscine toti-like viruses with genome characteristics including additional ORFs downstream of the classical capsid-RdRp ORFs (i.e., PMCV, GSTLV-1, CCTLV-1, and CLuTLV) seems to represent a discrete phylogenetic clade and based on their distinctive host range they appear to represent a distinct taxon. We suggest that this group is classified as a new genus, for which we propose the name “*Pistolvirus*” to reflect the grouping of the piscine toti-like viruses.

The remaining two piscine toti-like viruses, GSTLV-2 and BGTLV-1, are not included in this genus as they branch together with arthropod toti-like viruses (in suggested genus *Artivirus* [10,41]) and Beihai toti-like virus 4 from horseshoe crab, respectively. The branching of GSTLV-2 with viruses of suggested genus *Artivirus* is supported by the characteristics of an ORF1 with extended length compared to assigned members of *Totiviridae*, which they all share. The genomic sequence achieved for the novel BGTLV-1 here, is not expected to be complete in both 5′ and 3′ ends. BGTLV-1 shares approximately 50% identity on both ORF1 and ORF2 encoded proteins (Supplementary Tables S1 and S2) with Beihai toti-like virus 4, which is described with a long ORF1 encoding a polyprotein of size 1291 amino acids and no additional separate ORFs. Due to the close relation of BGTLV-1 to this virus, the genomic sequence presented for BGTLV-1 here is expected to be similar with an extended length of ORF1 encoding a polyprotein. In addition, lack of relation to the piscine viruses including additional ORFs in the 3′ end, indicates that the incomplete sequence information mainly relates to the 5′ end.

Questions have been raised if the new toti-like viruses are actually infecting the host they are discovered in or if they are viruses infecting a fungi or protozoa, like the registered members of *Totiviridae*, parasitizing the host. This is particularly relevant given the increasing number of novel viruses discovered in metatranscriptomics studies where analyses are purely based on sequence alone. This is of course theoretically possible, but for some viruses (e.g., PMCV and IMNV) histological studies have never indicated the presence of uni-cellular organisms in relation to disease lesions. Furthermore, in situ hybridization techniques have shown PMCV and IMNV specific RNA inside cells in lesions of CMS diseased salmon heart and IMN affected skeletal muscle of shrimp, respectively [9,14,49,50,51,52,53]. Moreover, the number of more diverse toti-like viruses found from advanced organisms are increasing, all carry additional genes which may be the result of an evolutionary achievement to compensate for more advanced mechanisms for infection and spread needed in such hosts. Phylogenetic studies also confirm the lack of close relation of toti-like viruses found in arthropods and piscine species to the genera of *Totiviridae*. Compared to viruses in the five genera of *Totiviridae*, all infecting uni-cellular hosts, the arthropod and piscine species must be the true hosts for the previous and recently described toti-like viruses. Still, short sequences from toti-like virus have been found in samples from both Atlantic salmon and Australasian snapper in metatranscriptomics studies in fish. The snapper virus was found to branch phylogenetically with genus *Victorivirus* and was expected to be a fungal virus [12]. The virus found on salmon shares no homology with PMCV, but is found with closest relation to Beihai blue swimmer crab virus 3 [13], more distant from the registered *Totiviridae* genera. The authors were not able to conclude if salmon was the true host of this virus. As mentioned above, several proofs show that PMCV is hosted by the Atlantic salmon. Similar, a piscine host is also expected for the five additional toti-like viruses described and compared in the present study since all these share genomic characteristics including additional genes and is by phylogeny not closely related to any of the genera of *Totiviridae*. Our results from RT-qPCR/NGS screening (details in Supplementary Text File S1) also confirm widespread distribution of the novel viruses in piscine species. Further, experimental studies should be conducted for experimental reproduction of disease for confirmation of significance of these novel viruses associated with mortality events, if directly associated with mortality events or indirectly, as part of the pathobiome.

The virus family *Totiviridae* is at present described to include isometric virions with no lipid or carbohydrate content reported and no surface projections. The virions contain a single segment of linear dsRNA, 4.6–7.0 kb in size enclosed in several copies of a single capsid protein of size 70–100kDa and a few copies of an RdRp protein are also included. The genome of viruses assigned to *Totiviridae* should have two large, usually overlapping, ORFs encoding the capsid and RdRp with the RdRp usually expressed as a C-terminal fusion to some copies of the capsid. These viruses are associated with latent infections of their fungal or protozoan hosts [1]. All of the more recently described toti-like viruses from arthropods, piscine, and planarian species share the majority of these characteristics and homology, especially related to the RdRp, indicating a relation to *Totiviridae*. Still, the fact that these viruses seems to include genomic sequences encoding more than the capsid and RdRp, which, for IMNV, includes a protein involved in virion surface projection, and that they are infecting much more advanced hosts than fungi and protozoa, probably using more advanced mechanisms, indicates that if included in the *Totiviridae*, the characteristics of the family have to be redefined.

## 5. Patents

Stian Nylund, Liv Sandlund and Arnfinn Økland, Pharmaq Analytiq, filed an application on parts of the research reported related to *Cyclopterus lumpus Toti-like virus* in this paper, under the European patent Office EP19155628 (International Application number PCT/EP2020/161105) “Novel Fish Totivirus”, May 2019. The invention relates to the novel virus, methods for detecting said virus and related use in a commercial setting.

## Figures and Tables

**Table 1:** Comparison of features of UTR’s and encoded proteins of the six piscine toti-like viruses. Number of amino acids (aa), predicted mass (kDa) and isoeletrict point (pI) of proteins encoded by the ORFs and GC content of nucleotides in each ORF. (**a**) General characteristics and (**b**) characteristics of full capsid-RdRp fusion protein if predicted -1 ribosomal frameshift is in use and fused RdRp-part from frameshift site in ORF1 to stop codon in ORF2.

(**a**)								
	**PMCV**	**GSTLV-1**	**CCTLV-1**	**CLuTLV**			**BGTLV-1**	**GSTLV-2**
**Total nt**	6688	6761	>5833 ^2^	6351			>5307 ^2,3^	7788 ^3^
**5′ UTR nt**	444	416	>64 ^2^	367			>192 ^2^	135
**3′ UTR nt**	241	235	>185 ^2^	132			>97 ^3^	>54 ^3^
**ORF 1/Capsid**								
**Peptides aa**	n.a ^4^	n.a ^4^	n.a ^4^	n.a ^4^			n.a ^4^	312 161 319
**Capsid aa**	861	852	>631 ^2^	828			891	867 ^3^
**Capsid kDa**	91.8	92.0	n.a. ^2,4^	90.5			n.a. ^2,4^	96.3
**pI of capsid**	5.4	5.8	n.a. ^2,4^	5.0			n.a. ^2,4^	5.47
**GC** (**%**)	56.2	51.4	n.a. ^2,4^	49.6			n.a. ^2,4^	37.2
**ORF2/RdRp**								
**aa**	726	725	640	647			785	737
**kDa**	83.1	94.8	73.5	74.2			88.9	85.8
**pI**	9.4	9.4	9.1	9.3			8.6	9.5
**GC** (**%**)	43.6	40.8	39.7	41.8			48.5	27.2
**ORF3**				ORF3	ORF4	ORF5		
**aa**	302	314 (338) ^1^	375	90	118	112	n.a ^4^	n.a ^4^
**kDa**	33.4	34.5 (37.3) ^1^	42.2	9.7	14.0	13.2	n.a ^4^	n.a ^4^
**pI**	7.0	9.2 (9.3) ^1^	6.5	5.2	6.1	4.4	n.a ^4^	n.a ^4^
**GC** (**%**)	45.0	42.1 (41.8) ^1^	44.3	48.5	46.3	39.9	n.a ^4^	n.a ^4^
(**b**)								
	**PMCV**	**GSTLV-1**	**CCTLV-1**	**CLuTLV**	**BGTLV-1**	**GSTLV-2**	**PMCV**	**GSTLV-1**
**Capsid-RdRp**								
**aa**	1616	1600	>1378 ^2^	1557	1673	1740	1616	1600
**kDa**	178.2	177.0	n.a. ^2,4^	174.2	n.a. ^2,4^	198.2	178.2	177.0
**pI**	8.9	8.0	n.a. ^2,4^	6.3	n.a. ^2,4^	8.8	8.9	8.0
**RdRp-part of fused**								
**aa**	757	750	749	729	782–785 ^5^	903	757	750
**kDa**	86.6	85.3	86.0	83.7	88.6–88.9	105.2	86.6	85.3
**pI**	9.6	9.0	8.8	9.3	8.6	9.3	9.6	9.0

^1^ Long alternative of GSTLV-1 ORF3 in brackets; ^2^ 5′ end of genome is incomplete, sizes might be inaccurate; ^3^ 3′end of genome is incomplete, sizes might be inaccurate; ^4^ n.a.—not applicable. ^5^ No defined frameshift site, size based on overlapping ORF1-ORF2.

## Data Availability

The complete nucleotide sequences achieved for GSTLV-1, CCTLV-1, BGTLV-1, and CLuTLV presented in this study are openly available in NCBI Genbank through accession numbers: MW888449 (GSTLV-1), MW893686 (CCTLV-1), MW893687 (BGTLV-1), and MW811138 (CLuTLV). Other data related to the presented in this study are available on request from the corresponding author.

## Supplementary Materials

The following are available online at https://www.mdpi.com/article/10.3390/v13061063/s1, Supplementary Text File S1: Additional information and results on origin of, prevalence and detection of GSTLV-1, CCTLV-1, BGTLV-1, and CLuTLV, Table S1: Overview of GenBank accession numbers for virus sequences used in Figure 3, Table S2: Similarity ORF1 of all viruses included in phylogenetic tree in Figure 6a, Table S3: Similarity ORF2 of all viruses included in phylogenetic tree in Figure 6b.
