# Supplementary material for: Comparative Molecular Characterization of Novel and Known Piscine Toti-Like Viruses
Source: Viruses. 2021 Jun 3;13(6):1063. doi: 10.3390/v13061063 (PMC8229945; doi:10.3390/v13061063)
Supplement: Supplementary file 1 [file viruses-13-01063-s001.zip › viruses-1200254-supplementary.pdf]

Supplementary Text file S1

Additional information and results on origin of, prevalence and detection of GSTLV-1, CCTLV-1, BGTLV-1 and CLuTLV

GSTLV-1

Origin of samples and virus

New whole genome sequencing and genomic analyses on GSTLV-1 (previously called golden shiner PMCV-like virus) was performed on an individual pool sample of five fish confirmed to have high viral loads using PCR analyses in our previous work [11]. Briefly, this sample originated in a study of golden shiners collected from retail baitshops located throughout Minnesota. Live fish were collected and delivered for diagnostic examination the same day to the University of Minnesota Veterinary Diagnostic Laboratory (UMVDL). At the UMVDL, kidney and spleen tissues were sampled, pooled and processed, and subjected to NGS analyses, from which the original partial genomic sequences of GSTLV-1 was identified and created the basis for the GSTLV-1 specific PCR used to identify the sample in this study.

CCTLV-1 and BGTLV-1

Origin of samples and virus and prescense of viral genomic sequences in species and lakes

Identification of viruses from common carp and bluegill, CCTLV-1 and BGTLV-1, originates in samples that belongs to a part of broad viral metagenomic study where dead fish were collected from natural mortality events in lakes distributed across the Upper Midwest region of the USA from June 2017 to October 2018. A total number of 22 bluegill tissue pools and 83 common carp tissue pools were included in the study. An overview of tissue pools and sampling location with positive detection of viral CCTLV-1 or BGTLV-1 sequences from dead wild fish in these lakes is shown in Table 1.

Table 1

Virus	Fish specie	Lake Name	Tissue pool	Result
**CCTLV-1**				
	Common Carp	Ballantyne	Kidney	Near complete genome
	Common Carp	Cottonwood	Brain	Partial genome
	Common Carp	Pamela	Brain	Partial genome
	Common Carp	Pamela	Gill	Partial genome
	Common Carp	Freeborn	Kidney	Partial genome
	Common Carp	Freeborn	Spleen	Partial genome
	Common Carp	Morehouse Park	Gill	Partial genome
	Common Carp	Morehouse Park	Kidney	Partial genome
	Common Carp	Morehouse Park	Spleen	Partial genome
	Common Carp	Currant	Kidney	Partial genome
	Common Carp	Currant	Spleen	Partial genome
	Common Carp	Sabre	Gill	Partial genome
	Common Carp	Sabre	Kidney	Partial genome
	Common Carp	Sabre	Spleen	Partial genome
**BGTLV-1**				
	Bluegill	Waconia	Spleen	Near complete genome
	Bluegill	Waconia	Gill	Partial genome

CLuTLV

Reverse transcription quantitative PCR screening for CLuTLV

Based on the sequencing results described in the main paper, PCR (described below) was performed on tissues (gills, heart, kidney, liver, spleen) as well as milt and eggs, collected from farmed adult lumpsuckers (n=933) along the coast of Norway as part of their routine screening pro-gram. In addition, samples from Atlantic salmon (n=133) and Ballan wrasse (n=12) were acquired from salmon farms along the coast of Norway as part of their regular screening program and screened by PCR. All CLuTLV-related samples were preserved on RNAlater ® (Sigma-Aldrich) at 4°C for appr. 24 hours before storage at -20°C until further procession.

Reverse transcription quantitative Real-time PCR (RT-qPCR) was performed and analyzed as described below using elongation factor 1 alpha (ef1α) from lumpsucker or Atlantic salmon as the internal control. Eight dilutions (2-fold) of RNA were used to create a standard curve and determine the efficiencies of the assays. All RT-qPCRs were carried out in triplicates. The tested assays were performed on RNA extracted from tissues from lumpsucker or salmon, simultaneously with ef1α using qScript XLT 1-step RT-qPCR Toughmix (Quantabio) following the manufacturer's protocol. Thermal cycling and detection/quantification were performed using Quantstudio™ 5 Real-time PCR system (Thermo Fisher Scientific) under the following conditions: cDNA synthesis; 50 °C; 15 min, enzyme activation; 95 °C, 2 min, followed by 40 cycles of denaturation of 95 °C, 5 s and extension; 55 °C; 45 s. Virus-specific primers used for RT-qRTPCR were as follows: CluTLV-F (5′ CTGTGGCTGTAAGTGCTCTAC-3′), CluTLV-R (5′ GCTGTTCTCTTTCCTCTCCTG-3′) and CluTLV-probe (5′ TTTCCGATGGTCATTGTGAACCTGG-3′).

A total of 922 samples from farmed lumpsucker from Norway have been screened for CLuTLV since first detection in March 2018 and up to January 2020. Samples including milt and eggs from broodstock, lumpsucker fry, and different tissue samples such as gills, liver, spleen and kidney tissues from adult fish, have been tested. Out of total number of samples, 157 tested positive for the virus resulting in a total prevalence of approximately 17%. Of the CLuTLV positive samples, the highest prevalence was found in lumpsucker fry (22%) and spleen (40%), liver (16%) and kidney (15%) from adult fish. CLuTLV has to this date not been found in any reproductive fluids.

Low levels of CLuTLV have also been detected in the kidney of one of 12 Ballan wrasse (*Labrus bergylta*) sampled in May 2018. In addition, 133 samples of Atlantic salmon including fry, smolt and broodstock that were screened for CLuTLV were negative for the virus.

Supplementary Table S1: Overview of Genbank accession numbers used for RdRp-motif comparison in Figure 3. RdRp-sequences from novel viruses described in manuscript is extracted from genomic sequence published in given accession number (marked by asterisks).

		Genbank accession
**PMCV**	Piscine myocarditis virus	ADP37187
**GSTLV-1**	golden shiner toti-like virus 1	MW893687*
**CCTLV-1**	common carp toti-like virus 1	MW893686*
**CLuTLV**	Cyclopterus lumpus toti-like virus	MW811138*
**ToVTJ**	Tianjin totivirus	YP_005454251
**OMRV**	Omono river virus	BAJ21511
**DTV**	drosophila totivirus	YP_003289293
**AsTV**	Armigeres subalbatus totivirus	ACH85916
**IMNV**	infectious myonecrosis virus	AAT6723
**GSTLV-2**	golden shiner toti-like virus 2	ANH79339
**Beihai toti-like 4**	Beihai toti-like virus 4	YP_009336713
**BGTLV-1**	bluegill toti-like virus 1	MW893687*
**Hubei toti-like 17**	Hubei toti-like virus 17	YP_009336917
**L. boulardi TLV**	Leptopilina boulardi toti-like virus	YP_009072448
**CYV**	Camponotus yamaokai virus	BAR72205
**GLV**	**Giardia lamblia virus**	**NP_620070**
**GCV**	Giardia canis virus	ABB36743
**LRV**	**Leishmania RNA virus**	**AAB50024**
**ScV-L-A**	**Saccharomyces cerevisiae virus L-A**	**AAA50321**
**ScV-L-BC (La)**	**Saccharomyces cerevisiae virus L-BC (La)**	**AAB02146**
**TVV1**	**Trichomonas vaginalis virus 1**	**AAA62868**
**TVV2**	Trichomonas vaginalis virus 2	AF127178_2
**Hv190SV**	Helminthosporium victoriae 190S virus	NP_619670

Table S2

Similarity ORF1 of all viruses included in phylogenetic tree in Figure 6a

	AME30134.1 [Piscine myocarditis-like virus]	**Golden shiner toti-like virus-1 (GSTLV-1)**	**Common carp toti-like virus-1 (CCTLV-1)**	AGA37431.1[Piscine myocarditis virus]	YP_004581249.1 [Piscine myocarditis virus AL V-708]	**Cyclopterus lupus toti-like virus (CLuTLV)**	**Bluegill toti-like virus-1 (BGTLV-1)**	YP_009336712.1[Beihai toti-like virus 4]	dd_Ptor_v3_38990_1_1[Planaria torva tricladivirus (PtorTV)]	GAKN01017247 [S. mediterranea¬†tricladivirus (SmedTV)]	NC_030295[Golden shiner toti-like virus-2 (GSTLV-2)]	YP_005454250.1[Tianjin totivirus]	BAJ21510.1[Omono River virus]	YP_003289292.1[Drosophila melanogaster totivirus SW-2009a]	ACH85915.1[Armigeres subalbatus virus SaX06-AK20]	AAT67230.1[Penaeid shrimp infectious myonecrosis virus]	BAR72204.1[Camponotus yamaokai virus]	AAF29444.1[Trichomonas vaginalis virus 2]	AAA62867.1[Trichomonas vaginalis virus 1]	AAA50320.1[Saccharomyces cerevisiae virus L-A]	AAA50506.1[Saccharomyces cerevisiae virus L-A]	AAB02145.1[Saccharomyces cerevisiae virus L-BC(La)]	YP_009072447.2[Leptopilina boulardi Toti-like virus]	NP_619669.2[Helminthosporium victoriae virus 190S]	AAB50030.1[Leishmania RNA virus 2]	AAB50023.1[Leishmania RNA virus 1]	ABB36742.1[Giardia canis virus]	AAM77693.1[Giardia lamblia virus]
AME30134.1 [Piscine myocarditis-like virus]		100	62.18	76.78	76.54	64.68	36.91	34.52	31.87	26.69	27.28	31.57	31.57	30.48	30.37	29.2	31.57	28.42	26.7	25.03	24.92	26.52	29.54	22.16	19.2	20.47	30.63	30.94
**Golden shiner toti-like virus-1 (GSTLV-1)**	100		61.02	76.42	76.19	63.67	35.71	27.37	23.55	18.87	17.68	20.57	20.52	26.26	22.22	28.34	31.4	29.27	26.58	25.39	25.28	27.01	20.97	20.39	18.32	19.23	29.4	29.71
**Common carp toti-like virus-1 (CCTLV-1)**	62.18	61.02		52.47	52.58	44.72	25.86	20.28	16.37	12.51	13.87	14.91	14.91	19.68	16.5	21.24	24.31	20.7	22.12	23.47	23.35	21.52	16.38	13.62	11.97	12.07	21.24	21.35
AGA37431.1[Piscine myocarditis virus]	76.78	76.42	52.47		99.77	62.38	34.83	27.42	23.13	18.83	18.01	20.09	20.03	25.76	22.26	28.5	32.36	28.9	25.82	25.72	25.51	24.84	20.59	22.05	18.32	18.14	29.24	29.17
YP_004581249.1 [Piscine myocarditis virus AL V-708]	76.54	76.19	52.58	99.77		62.5	34.93	27.35	23.19	18.83	18.07	20.03	19.98	25.91	22.32	28.79	32.27	28.79	25.93	25.61	25.41	24.84	20.59	22.14	18.14	17.96	29.33	29.27
**Cyclopterus lupus toti-like virus (CLuTLV)**	64.68	63.67	44.72	62.38	62.5		34.38	28.21	22.15	18.7	17.94	18.82	18.82	23.91	21.54	29.39	30.6	30.29	27.2	23.54	23.44	28.54	19.93	21.49	17.63	17.91	28.44	28.37
**Bluegill toti-like virus-1 (BGTLV-1)**	36.91	35.71	25.86	34.83	34.93	34.38		34.44	24.14	20.14	18.6	18.38	18.38	24.64	20.89	30.21	33.39	27.59	24.29	22.08	21.89	26.24	20.33	19.38	16.65	16.03	26.02	26.2
YP_009336712.1[Beihai toti-like virus 4]	34.52	27.37	20.28	27.42	27.35	28.21	34.44		32.7	30.96	28.38	27.67	27.51	31.35	28.96	23.16	27.37	20.64	22.14	18.63	18.63	20.42	29.22	23.05	20.81	20.52	26.35	26.5
dd_Ptor_v3_38990_1_1[Planaria torva tricladivirus (PtorTV)]	31.87	23.55	16.37	23.13	23.19	22.15	24.14	32.7		45.08	30.24	30.66	30.46	28.17	31.47	20	24.87	16.46	18.4	18.41	18.47	17.94	31.9	19.07	17.03	17.44	22.3	22.47
GAKN01017247 [S. mediterranea¬†tricladivirus (SmedTV)]	26.69	18.87	12.51	18.83	18.83	18.7	20.14	30.96	45.08		27.91	27.49	27.43	23.88	27.25	15.21	18.26	15.34	17.73	16.73	16.67	14.88	29.47	20.61	19.29	19.47	19.03	19.03
NC_030295[Golden shiner toti-like virus-2 (GSTLV-2)]	27.28	17.68	13.87	18.01	18.07	17.94	18.6	28.38	30.24	27.91		50.72	50.61	45.45	50.87	31.67	19.99	16.09	15.57	16.58	16.52	16.25	28.75	18.12	15.94	17.05	20.38	20.49
YP_005454250.1[Tianjin totivirus]	31.57	20.57	14.91	20.09	20.03	18.82	18.38	27.67	30.66	27.49	50.72		99.82	58.65	65.37	32.77	20.01	16.7	15.54	16.22	16.16	18.11	31.3	17.68	15.63	15.55	21.13	21.13
BAJ21510.1[Omono River virus]	31.57	20.52	14.91	20.03	19.98	18.82	18.38	27.51	30.46	27.43	50.61	99.82		58.65	65.43	32.77	20.07	16.7	15.71	16.22	16.16	18.05	31.25	17.74	15.75	15.55	20.91	20.91
YP_003289292.1[Drosophila melanogaster totivirus SW-2009a]	30.48	26.26	19.68	25.76	25.91	23.91	24.64	31.35	28.17	23.88	45.45	58.65	58.65		59.72	42.46	25.43	23.24	21.62	21.99	21.83	23.18	27.71	23.83	21.65	20.94	26.32	26.49
ACH85915.1[Armigeres subalbatus virus SaX06-AK20]	30.37	22.22	16.5	22.26	22.32	21.54	20.89	28.96	31.47	27.25	50.87	65.37	65.43	59.72		37.53	21.91	18.99	17.92	18.92	18.85	19.79	31.37	20.29	17.8	17.7	23.05	23.19
AAT67230.1[Penaeid shrimp infectious myonecrosis virus]	29.2	28.34	21.24	28.5	28.79	29.39	30.21	23.16	20	15.21	31.67	32.77	32.77	42.46	37.53		27.92	25.22	18.96	24.52	24.71	24.81	19.83	16.49	14.74	15.35	25.35	25.55
BAR72204.1[Camponotus yamaokai virus]	31.57	31.4	24.31	32.36	32.27	30.6	33.39	27.37	24.87	18.26	19.99	20.01	20.07	25.43	21.91	27.92		22.85	24.5	22.39	22.3	23.64	20.47	19.95	17.75	17.03	27.74	27.94
AAF29444.1[Trichomonas vaginalis virus 2]	28.42	29.27	20.7	28.9	28.79	30.29	27.59	20.64	16.46	15.34	16.09	16.7	16.7	23.24	18.99	25.22	22.85		22.97	23.51	23.62	24.19	16.28	17.29	16.15	16.51	24.56	24.78
AAA62867.1[Trichomonas vaginalis virus 1]	26.7	26.58	22.12	25.82	25.93	27.2	24.29	22.14	18.4	17.73	15.57	15.54	15.71	21.62	17.92	18.96	24.5	22.97		24.36	24.58	23.85	16.84	25.91	20.84	20.75	26.04	26.37
AAA50320.1[Saccharomyces cerevisiae virus L-A]	25.03	25.39	23.47	25.72	25.61	23.54	22.08	18.63	18.41	16.73	16.58	16.22	16.22	21.99	18.92	24.52	22.39	23.51	24.36		99.85	25.38	14.79	19.38	18.06	18.57	27.2	27.13
AAA50506.1[Saccharomyces cerevisiae virus L-A]	24.92	25.28	23.35	25.51	25.41	23.44	21.89	18.63	18.47	16.67	16.52	16.16	16.16	21.83	18.85	24.71	22.3	23.62	24.58	99.85		25.38	14.68	19.48	18.06	18.57	27.1	27.03
AAB02145.1[Saccharomyces cerevisiae virus L-BC(La)]	26.52	27.01	21.52	24.84	24.84	28.54	26.24	20.42	17.94	14.88	16.25	18.11	18.05	23.18	19.79	24.81	23.64	24.19	23.85	25.38	25.38		17.27	17.83	17.37	19.82	24.98	24.48
YP_009072447.2[Leptopilina boulardi Toti-like virus]	29.54	20.97	16.38	20.59	20.59	19.93	20.33	29.22	31.9	29.47	28.75	31.3	31.25	27.71	31.37	19.83	20.47	16.28	16.84	14.79	14.68	17.27		17.24	15.15	15.2	19.61	19.67
NP_619669.2[Helminthosporium victoriae virus 190S]	22.16	20.39	13.62	22.05	22.14	21.49	19.38	23.05	19.07	20.61	18.12	17.68	17.74	23.83	20.29	16.49	19.95	17.29	25.91	19.38	19.48	17.83	17.24		33.67	34.17	19.96	19.7
AAB50030.1[Leishmania RNA virus 2]	19.2	18.32	11.97	18.32	18.14	17.63	16.65	20.81	17.03	19.29	15.94	15.63	15.75	21.65	17.8	14.74	17.75	16.15	20.84	18.06	18.06	17.37	15.15	33.67		66.36	18.82	18.84
AAB50023.1[Leishmania RNA virus 1]	20.47	19.23	12.07	18.14	17.96	17.91	16.03	20.52	17.44	19.47	17.05	15.55	15.55	20.94	17.7	15.35	17.03	16.51	20.75	18.57	18.57	19.82	15.2	34.17	66.36		17.62	17.72
ABB36742.1[Giardia canis virus]	30.63	29.4	21.24	29.24	29.33	28.44	26.02	26.35	22.3	19.03	20.38	21.13	20.91	26.32	23.05	25.35	27.74	24.56	26.04	27.2	27.1	24.98	19.61	19.96	18.82	17.62		98.87
AAM77693.1[Giardia lamblia virus]	30.94	29.71	21.35	29.17	29.27	28.37	26.2	26.5	22.47	19.03	20.49	21.13	20.91	26.49	23.19	25.55	27.94	24.78	26.37	27.13	27.03	24.48	19.67	19.7	18.84	17.72	98.87	

Table S3

Similarity ORF2 of all viruses included in phylogenetic tree in Figure 6b

	AFQ55381.1[Saccharomyces cerevisiae virus L-A-lus]	AHF27234.1[Saccharomyces cerevisiae virus L-A-2]	NP_620493.1[Saccharomyces cerevisiae virus L-A]	NP_619670.2[Helminthosporium victoriae virus 190S]	AAB50031.1[Leishmania RNA virus 2]	AAB50024.1[Leishmania RNA virus 1]	AAF29445.1[Trichomonas vaginalis virus 2]	AAA62868.1[Trichomonas vaginalis virus 1]	AME30135.1 [Piscine myocarditis-like virus]	Golden shiner toti-like virus-1 (GSTLV-1)	Common carp toti-like virus-1 (CCTLV-1)	YP_004581250.1 [Piscine myocarditis virus AL V-708]	Cyclopterus lupus toti-like virus (CLuTLV)	YP_009336917.1[Hubei toti-like virus 17]	YP_009072448.1[Leptopilina boulardi Toti-like virus]	YP_009336713.1[Beihai toti-like virus 4]	Bluegill toti-like virus-1 (BGTLV-1)	ABB36743.1[Giardia canis virus]	AAM77694.1[Giardia lamblia virus]	dd_Ptor_v3_38990_1_1[Planaria torva tricladivirus (PtorTV)]	GAKN01017247 [S. mediterranea¬†tricladivirus (SmedTV)]	BAR72205.1[Camponotus yamaokai virus]	NC_030295[Golden shiner toti-like virus-2 (GSTLV-2)]	YP_005454251.1[Tianjin totivirus]	BAJ21511.1[Omono River virus]	YP_003289293.1[Drosophila melanogaster totivirus SW-2009a]	ACH85916.1[Armigeres subalbatus virus SaX06-AK20]	AAT67231.2[Penaeid shrimp infectious myonecrosis virus]
AFQ55381.1[Saccharomyces cerevisiae virus L-A-lus]		98.27	96.08	25.47	22.9	26.65	23.67	23.01	23.35	29.31	22.67	18.9	16.88	18.08	19.92	21.59	18.76	36.22	20.3	20.98	25.36	17.82	19.1	41.19	20.04	45.83	18.4	19.73
AHF27234.1[Saccharomyces cerevisiae virus L-A-2]	98.27		95.88	25.54	23.03	26.52	23.48	23.14	22.96	28.82	22.41	18.77	16.69	18.41	20.18	21.91	18.76	36.22	20.19	20.6	25.36	17.43	19.16	40.78	19.78	45.49	17.82	19.54
NP_620493.1[Saccharomyces cerevisiae virus L-A]	96.08	95.88		25.6	23.55	26.39	23.54	23.27	23.15	29.06	22.41	18.96	16.49	18.08	20.44	21.65	18.76	36.12	20.07	21.23	25.3	17.69	19.29	40.22	19.53	44.44	18.2	19.35
NP_619670.2[Helminthosporium victoriae virus 190S]	25.47	25.54	25.6		50.92	52.34	39.89	38.39	38.24	39.16	37.31	34.59	34.02	29.96	33.66	35.87	33.59	20.52	30.16	33.26	34.09	29.78	29.74	37.31	32.8	37.62	30.42	31.09
AAB50031.1[Leishmania RNA virus 2]	22.9	23.03	23.55	50.92		61.97	44.25	42.37	39.55	39.48	37.77	36.92	36.02	34.5	34.87	39.14	36.28	18.28	30.9	35.19	32.03	34.79	34.13	36.52	34.47	38.07	34.85	35.46
AAB50024.1[Leishmania RNA virus 1]	26.65	26.52	26.39	52.34	61.97		44.91	44.24	40.55	42.71	37.91	33.08	31.92	29.84	30.73	34.38	33.44	21.91	28.63	32.35	34.17	29.74	30.53	36.62	30.57	38.92	29.94	30.07
AAF29445.1[Trichomonas vaginalis virus 2]	23.67	23.48	23.54	39.89	44.25	44.91		49.82	34.06	35.8	35.38	31.38	28.64	28.52	30.01	31.7	31.73	18.55	26.45	31.12	33.05	28.89	29.94	33.29	28.45	33.97	27.78	28.92
AAA62868.1[Trichomonas vaginalis virus 1]	23.01	23.14	23.27	38.39	42.37	44.24	49.82		34.87	36.38	34.23	30.81	30.83	33.84	33.79	37.03	35.47	20.21	30.71	32.87	33.59	33.81	32.37	35.26	31.22	38.31	31.05	33.78
AME30135.1 [Piscine myocarditis-like virus]	23.35	22.96	23.15	38.24	39.55	40.55	34.06	34.87		100	76.79	60.14	54.6	39.14	36.25	42.6	38.23	22.15	34.98	39.46	38.45	35.02	32.88	39.57	34	39.66	33.05	34.64
Golden shiner toti-like virus-1 (GSTLV-1)	29.31	28.82	29.06	39.16	39.48	42.71	35.8	36.38	100		76.77	60.09	54.55	39.29	36.22	42.54	38.2	29.39	34.92	39.4	40.58	34.94	32.81	39.57	33.93	39.66	32.98	34.57
Common carp toti-like virus-1 (CCTLV-1)	22.67	22.41	22.41	37.31	37.77	37.91	35.38	34.23	76.79	76.77		61.12	54	39.03	36.94	43.07	38.13	21.8	34.24	38.03	39.13	35.93	32.15	39.64	34.39	39.3	32.63	34.64
YP_004581250.1 [Piscine myocarditis virus AL V-708]	18.9	18.77	18.96	34.59	36.92	33.08	31.38	30.81	60.14	60.09	61.12		62.5	42.59	39.08	42.71	40.91	18.55	33.24	38.17	34.22	41.77	36.72	36.44	35.08	38.28	36	38.88
Cyclopterus lupus toti-like virus (CLuTLV)	16.88	16.69	16.49	34.02	36.02	31.92	28.64	30.83	54.6	54.55	54	62.5		45.71	38.79	42.52	42.06	16.72	30.09	35.29	31.44	44.77	40.08	37.76	36.07	39.08	36.7	40.59
YP_009336917.1[Hubei toti-like virus 17]	18.08	18.41	18.08	29.96	34.5	29.84	28.52	33.84	39.14	39.29	39.03	42.59	45.71		41.67	44.85	42.72	18.99	33.43	40.83	34.32	40.43	39.95	38.4	36.54	39.35	38.61	40.15
YP_009072448.1[Leptopilina boulardi Toti-like virus]	19.92	20.18	20.44	33.66	34.87	30.73	30.01	33.79	36.25	36.22	36.94	39.08	38.79	41.67		42.52	43.04	18.33	32.3	40.22	33.3	34.58	38.34	41.27	40.71	43.58	40.57	40.73
YP_009336713.1[Beihai toti-like virus 4]	21.59	21.91	21.65	35.87	39.14	34.38	31.7	37.03	42.6	42.54	43.07	42.71	42.52	44.85	42.52		50.17	20.55	35.44	46.62	40.76	39.95	39.19	41.97	40.36	43.07	40.79	42.99
Bluegill toti-like virus-1 (BGTLV-1)	18.76	18.76	18.76	33.59	36.28	33.44	31.73	35.47	38.23	38.2	38.13	40.91	42.06	42.72	43.04	50.17		18.29	32.38	42.7	37.19	37.03	36.48	38.95	38.77	40.09	38.78	39.37
ABB36743.1[Giardia canis virus]	36.22	36.22	36.12	20.52	18.28	21.91	18.55	20.21	22.15	29.39	21.8	18.55	16.72	18.99	18.33	20.55	18.29		54.94	19.26	24.16	16.53	16.13	33.6	18.18	37.69	17.19	17.35
AAM77694.1[Giardia lamblia virus]	20.3	20.19	20.07	30.16	30.9	28.63	26.45	30.71	34.98	34.92	34.24	33.24	30.09	33.43	32.3	35.44	32.38	54.94		34.06	30.95	29.69	28.34	33.6	32.09	37.69	30.42	30.51
dd_Ptor_v3_38990_1_1[Planaria torva tricladivirus (PtorTV)]	20.98	20.6	21.23	33.26	35.19	32.35	31.12	32.87	39.46	39.4	38.03	38.17	35.29	40.83	40.22	46.62	42.7	19.26	34.06		60.1	32.93	36.44	41.3	39.48	44.01	36.5	39.2
GAKN01017247 [S. mediterranea¬†tricladivirus (SmedTV)]	25.36	25.36	25.3	34.09	32.03	34.17	33.05	33.59	38.45	40.58	39.13	34.22	31.44	34.32	33.3	40.76	37.19	24.16	30.95	60.1		28.34	30.85	41.57	33.74	42.9	31.02	33.83
BAR72205.1[Camponotus yamaokai virus]	17.82	17.43	17.69	29.78	34.79	29.74	28.89	33.81	35.02	34.94	35.93	41.77	44.77	40.43	34.58	39.95	37.03	16.53	29.69	32.93	28.34		38.53	38.81	37.44	38.52	39.04	39.51
NC_030295[Golden shiner toti-like virus-2 (GSTLV-2)]	19.1	19.16	19.29	29.74	34.13	30.53	29.94	32.37	32.88	32.81	32.15	36.72	40.08	39.95	38.34	39.19	36.48	16.13	28.34	36.44	30.85	38.53		68.51	65.29	70.57	64.99	68.97
YP_005454251.1[Tianjin totivirus]	41.19	40.78	40.22	37.31	36.52	36.62	33.29	35.26	39.57	39.57	39.64	36.44	37.76	38.4	41.27	41.97	38.95	33.6	33.6	41.3	41.57	38.81	68.51		100	84.24	75.47	73.04
BAJ21511.1[Omono River virus]	20.04	19.78	19.53	32.8	34.47	30.57	28.45	31.22	34	33.93	34.39	35.08	36.07	36.54	40.71	40.36	38.77	18.18	32.09	39.48	33.74	37.44	65.29	100		84.08	73	69.57
YP_003289293.1[Drosophila melanogaster totivirus SW-2009a]	45.83	45.49	44.44	37.62	38.07	38.92	33.97	38.31	39.66	39.66	39.3	38.28	39.08	39.35	43.58	43.07	40.09	37.69	37.69	44.01	42.9	38.52	70.57	84.24	84.08		76.67	73.03
ACH85916.1[Armigeres subalbatus virus SaX06-AK20]	18.4	17.82	18.2	30.42	34.85	29.94	27.78	31.05	33.05	32.98	32.63	36	36.7	38.61	40.57	40.79	38.78	17.19	30.42	36.5	31.02	39.04	64.99	75.47	73	76.67		72.1
AAT67231.2[Penaeid shrimp infectious myonecrosis virus]	19.73	19.54	19.35	31.09	35.46	30.07	28.92	33.78	34.64	34.57	34.64	38.88	40.59	40.15	40.73	42.99	39.37	17.35	30.51	39.2	33.83	39.51	68.97	73.04	69.57	73.03	72.1	
